# Future Directions for Clinical Respiratory Fungal Research

**DOI:** 10.1007/s11046-021-00579-5

**Published:** 2021-09-29

**Authors:** Darius Armstrong-James

**Affiliations:** Department of Infectious Diseases, Imperial College London, London, UK

**Keywords:** Respiratory, Fungal, Infection, Allergy, Antifungals, Immunotherapy, Personalized medicine

## Abstract

There has been a growing appreciation of the importance of respiratory fungal diseases in recent years, with better understanding of their prevalence as well as their global distribution. In step with the greater awareness of these complex infections, we are currently poised to make major advances in the characterization and treatment of these fungal diseases, which in itself is largely a consequence of post-genomic technologies which have enabled rational drug development and a path towards personalized medicines. These advances are set against a backdrop of globalization and anthropogenic change, which have impacted the world-wide distribution of fungi and antifungal resistance, as well as our built environment. The current revolution in immunomodulatory therapies has led to a rapidly evolving population at-risk for respiratory fungal disease. Whilst challenges are considerable, perhaps the tools we now have to manage these infections are up to this challenge. There has been a welcome acceleration of the antifungal pipeline in recent years, with a number of new drug classes in clinical or pre-clinical development, as well as new focus on inhaled antifungal drug delivery. The “post-genomic” revolution has opened up metagenomic diagnostic approaches spanning host immunogenetics to the fungal mycobiome that have allowed better characterization of respiratory fungal disease endotypes. When these advances are considered together the key challenge is clear: to develop a personalized medicine framework to enable a rational therapeutic approach.

## Introduction

Respiratory fungal diseases have risen in prominence in recent years, as a consequence of improved diagnostics, advocacy, research and greater awareness [1–6]. However, our understanding of whether there has been a genuine increase in the prevalence of respiratory fungal diseases is less clear. Current advances across a range of different areas, including immunophenotyping, metagenomics, antifungal therapies and immunotherapies have opened up exciting opportunities to revolutionize our clinical approach to these complex respiratory infections.

### Opportunistic Respiratory Mycoses

Fungal opportunism of the respiratory tract has been dominated by the aspergilli, and in particular *Aspergillus fumigatus* [7]*. Aspergillosis* was first described in humans by Dieulafoy in the 1890s as a primary pulmonary infection, and as fungal rhinitis in 1915 [8, 9]. The ubiquitous global nature of this saprophytic mould, with small, highly dispersible conidia which are inhaled on a daily basis, and its thermophilic nature make it ideally suited as a pulmonary pathogen [7, 10]. Other species within the genus have played a prominent role as respiratory mycoses [11, 12]. Within the broader context of allergic fungal airway disease, fungal sensitization may be mediated by thermophilic fungi such as *Aspergillus* spp., and *Candida* spp. as well as thermo-intolerant fungi such as the *Cladosporium* and *Alternaria* genera [13, 14]. In the immunocompromised host, a much broader range of opportunistic fungi, such as *Pneumocystis jirovecii*, the mucoromycotina, and *Cryptococcus spp.* may cause invasive infection [15–17]. Fungi are also prominent causative agents of hypersensitivity pneumonitis, implicated in farmer’s lung disease (*Aspergillus fumigatus*, *Lichtheimia corymbifera*), and peat moss exposure (*Penicillium* spp.) amongst others [18, 19]. Thus, fungi are remarkable in their ability to induce both invasive infections as well as allergic sensitization and hypersensitivity responses.

### Endemic Respiratory Mycoses

Endemic respiratory mycoses are characterized as primary pathogens that can also disseminate, often in the context of immunocompromised [20]. Histoplasmosis, an endemic mycosis of the Americas and opportunistic mycosis globally (var. *duboisii*), was first described as a human pathogen by Darling (Darling’s disease) in 1909 [21]. Talaromycosis*,* an East Asian endemic mycosis due to *Talaromyces marneffei* (formerly *Penicillium marneffei*),, was first described in 1959 by Segretain [22], and coccidioidomycosis, due to *Coccidioides immitis*, a mycoses of the Americas, was first described in 1948 [23, 24]. Blastomycosis, an American endemic mycosis due to *Blastomyces dermatitidis* was first described in 1914 [25]. Endemic mycosis gained increasing prominence, mainly as a consequence of their association with the AIDS epidemic, where they are major opportunistic pathogens, from the 1980s onwards [26]. The current revolution in immunomodulatory therapies has led to new patient groups becoming at risk of endemic mycoses [27].

### Epidemiology

The epidemiological drivers of respiratory fungal disease are complex and contingent on a range of factors, many of which may have an anthropogenic basis [28]. For instance, global warming and the global trade in plants may have rapidly accelerated the propensity for new and ecologically invasive species, as well as the rapid global emergence of triazole resistance, recently characterized in *A. fumigatus* [29]. Changes in building construction and in particular ventilation may have led to fundamental shifts in the composition and diversity of the aerial mycobiota in the built environment [30]. Susceptibility to fungal infection is increasingly complex, where novel agents such as ibrutinib and IL-5 modulators have been shown to have potential to predispose individuals to respiratory fungal infection [31]. The widespread use of steroids is thought to have played a major role in the apparent increasing incidence of chronic respiratory fungal diseases in the context of chronic diseases of the lung [32, 33].

## How Can We Use Available and Emerging Antifungals to Improve Outcomes from Respiratory Fungal Disease?

Much of our current understanding around the optimal use of antifungals for respiratory fungal diseases has been driven by well-funded, commercial, randomized controlled studies in the context of invasive pulmonary aspergillosis in the immunocompromised host [34, 35]. This has led to the licensing of voriconazole, ambisome, posaconazole and isavuconazole in this setting [34–37]. However, our understanding of how these agents can be used in the context of chronic respiratory fungal disease is less well defined [38, 39]. Most clinical trial data are centred around itraconazole, a historical azole with poor oral absorption, major drug interactions and significant side effects [40]. Furthermore, historical studies in invasive pulmonary aspergillosis have already established that itraconazole has poor efficacy for invasive aspergillosis, and therefore likely to be inferior to newer mould-active triazoles [41]. It is therefore not surprising that studies of itraconazole therapy in the context of chronic pulmonary aspergillosis, allergic bronchopulmonary aspergillosis and severe asthma with fungal sensitization have by and large showed modest or no effect. In addition, the importance of triazole therapeutic drug monitoring, which has not been addressed in clinical trials, but is now de facto standard of care in the real world, is a major confounder for these studies that requires further exploration. It is notable that those studies that failed did not undertake therapeutic drug monitoring, which is not currently a requirement under the licensing of any triazole antifungal [42]. The only study to address voriconazole in the context of allergic fungal airway disease, EVITA3, also did not involve therapeutic drug monitoring [43]. Furthermore, whilst therapy was stopped at 3 months, clinical endpoint measurements were undertaken at 12 months, on the basis that any antifungal effect would be long-lived. However, unlike itraconazole, voriconazole is aquaphilic and does not persist in tissues in the long-term. Thus, further studies of newer triazoles such as voriconazole, posaconazole and isavuconazole in the context of chronic respiratory fungal disease are urgently needed.

There is currently a lack of clarity around the use of antifungals for allergic fungal airway diseases such as severe asthma with fungal sensitization and allergic bronchopulmonary aspergillosis, where historically it was thought that these fungal diseases are purely driven by sensitization to the aerial mycobiota rather than any element of airway infection. This theory seems reasonable in the context of thermointolerant fungi such as *Alternaria* spp.; however for allergic bronchopulmonary aspergillosis the presence of hyphae in the mucous and evidence of mucosal inflammation with a positive *Aspergillus* IgG response in serum are suggestive of airway mycosis [44]. Moreover, recent careful mycological studies suggest the involvement of airway mycosis in allergic airway disease could be much more extensive than is currently believed [45]. Better understanding of these relationships and the role that antifungals could play are urgently needed.

We are currently in the midst of a revolution in the antifungal armamentorium, with posaconazole and isavuconazole representing a step change for triazole usage clinically, as a consequence of their improved side effect profiles, absorbance and spectrum of action [34, 46–49]. A number of exciting new drug classes are now in late phase clinical studies, and there has been renewed interest in inhaled antifungal development. Olorofim (F901318; F2G Ltd., Manchester, UK) is the first of a new class of drugs, the orotomides, that inhibit dihydroorotate dehydrogenase, a key enzyme in pyrimidine synthesis [50]. The drug is available orally and intravenously with wide tissue distribution. Notably the drug has a wide spectrum of activity against *Aspergillus* spp., including triazole-resistant strains, as well as more difficult to treat opportunistic pulmonary fungal pathogens such as *Lomentospora prolificans* and *Scedosporium* spp. as well as the causative agents of endemic mycoses [51–54]. However, there is a lack of activity against *Candida* spp., mucoralean fungi, and *Cryptococcus* spp. There is an open label study ongoing to evaluate the utility of olorofim in individuals with limited treatment options (FORMULA; NTC03583164).

Fosmanogepix (APX001; Amplyx, San Diego, Ca.), is a prodrug metabolized to manogepix, its active form [55]. It disrupts glycosylphosphatidylinositol (GPI)-anchor biosynthesis by inhibiting the enzyme Gwt1 and has good activity in vitro against *Aspergillus* spp., *Cryptococcus neoformans*, *Scedosporium* spp., and *Fusarium* spp. [56, 57]. There is currently a phase 2, multicentre study to evaluate Fosmanogepix for the treatment of invasive fungal infections caused by *Aspergillus* spp. or rare moulds (e.g. *Scedosporium* spp., *Fusarium* spp., and mucoralean fungi).

A number of other systemic agents are in various stages of development that could be useful in the setting of respiratory fungal disease such as orally available amphotericin B Cochleate (CAMB/MAT2203; Matinas Biopharma, Bedminster, NJ) [58, 59], MGCD290 (Mirati Therapeutics, San Diego, Ca.) [60, 61], tetrazoles (VT-1129, VT-1161, and VT-1598; Viamet Pharmaceuticals, Durham, NC) [62–67], VL-2397/ASP2397 (Vical inc.; San Diego, Ca.) [68], and T-2307 (Toyama Chemical, Tokyo, Japan) [69].

Inhaled antifungals represent a particularly interesting area for development in the context of respiratory fungal disease, where there is potential to achieve increased concentrations of drug in the respiratory mucosa compared to the systemic route, and the possibility for synergies with systemic agents. Historically amphotericin B has been used both as deoxycholate as well as in lipid forms, primarily as nebulized prophylaxis against pulmonary mould infection in haematological malignancies, and lung transplantation [70–73]. Another setting is as therapy for allergic bronchopulmonary aspergillosis or *Aspergillus* tracheobronchitis [70, 74]. There have been case reports around the use of nebulized triazoles for the treatment of airway mycoses with varying success. More recently there has been a concerted effort to systematically develop specifically formulated nebulized antifungals with good airway distribution and retention. PC945 (Pulmocide Ltd.) is a novel triazole specifically designed to achieve high concentrations in the airway mucosa with limited systemic exposure [75]. It has potent activity against *Aspergillus* and accumulates in the lung on repeat dosing [76]. Interestingly animal studies indicate improved efficacy when combined with systemic antifungals in murine pulmonary aspergillosis [77, 78]. It was well tolerated in healthy individuals and asthmatics. Initial case reports for nebulized PC945 as salvage therapy in refractory lung transplant *Aspergillus* tracheobronchitis showed complete response [75, 79, 80]. Phase 2 study data in asthma and cystic fibrosis patients with pulmonary aspergillosis are currently being evaluated. Pulmazole (Pulmatrix Inc) is a new dry powder itraconazole formulation that was being evaluated in adult asthmatics with allergic bronchopulmonary aspergillosis in Phase 2 studies (*NCT03960606*). Pulmazole is engineered using propriety technology that allows particles to be formulated as small, dense and dispersible particles for deep lung penetration. This allows for delivery as a dry powder by inhalation. There are also two formulations of voriconazole in development for inhalation, ZP-059 (Zambon Company S.P.A., Milano, Italy), and TFF-VORI (TFF pharmaceuticals, Austin, TX) that have completed Phase 1 of development.

Taken together, the likely availability of novel systemic antifungal drug classes as well as the option for inhalational antifungals has the potential to dramatically change the clinical landscape for therapeutic options for respiratory fungal diseases. A particularly exciting challenge will be to work out if combination therapies are superior and in particular whether the combination of systemic and inhaled antifungals is superior to conventional systemic therapies that are currently prevalent. This is particularly important in the context of airway mycoses where it seems likely that current systemic antifungal treatments are sup-optimal. Another unanswered question is around what utility adjunctive inhaled antifungals could have in the context of invasive pulmonary mycoses such as invasive aspergillosis. Finally, there are major unanswered questions around duration of therapy, with most trials of invasive aspergillosis using 6–12 weeks therapy but very little data to guide where shorter courses may be appropriate.

## How Can We Improve the Diagnosis of Respiratory Fungal Infection?

Current diagnosis of respiratory fungal diseases revolves around three central pillars, the mycological evidence of infection, the clinical status of the host and the evidence that there is an immune response to a fungus in the host.

### Detection of Fungal Infection

Classically mycological criteria have typically been from fungal cultures from either the airway or on tissue biopsy; however these would not necessarily be diagnostic on their own (unless for an endemic mycoses) as the airway has always been considered non-sterile from a microbiological perspective and fungi are ubiquitous components of the aerial microbiota [81]. Fungal polymerase chain reaction has been available for several decades, has long been established for the diagnosis of *Pneumocystis* pneumonia and has recently been approved for the diagnosis of invasive pulmonary aspergillosis [81]. There has been limited work on the utility of fungal species multiplex PCRs, which would be highly attractive for airway samples across a range of settings such as haematological immunocompromised, lung transplantation and cystic fibrosis where a specific, limited group of fungal pathogens account for the vast majority of infections [82, 83]. Further progress has been made with respect to more systematic use of both β-1,3 glucan and galactomannan as markers of respiratory fungal disease where β-1,3 glucan is used in serum primarily as a screening assay and galactomannan has utility both in serum and airway samples for specific diagnosis of aspergillosis. There has been significant advance in the availability of lateral flow device assays for point-of-care testing across aspergillosis as well as endemic mycoses such as histoplasmosis [84, 85]. A further area of ongoing development is whether urinary antigens have utility for the diagnosis of respiratory mycoses [86–88]. In general terms, it seems clear that the combination of two different assays such as PCR and antigen for identification of fungal disease leads to a much more robust diagnostic performance profile. The elephant in the room in terms of mycological diagnosis of respiratory mycoses is the airway mycobiome. There has been a significant body of work to describe this across a range of settings from the immunocompromised host, where it has been shown the mycobiome or even the microbiome could predict pulmonary fungal disease [89–91]. However, this work is still at a very early phase [92]. Further detailed studies have been undertaken in chronic respiratory fungal diseases as well as in chronic respiratory disease more generally, to determine either the role that fungi play in the pathogenesis of diseases such as asthma, COPD and bronchiectasis, or to delineate the composition of the mycobiome and microbiome underlying conditions such as allergic bronchopulmonary aspergillosis or cystic fibrosis-related aspergillosis [93–95]. Bigger multicentre studies using the latest metagenomic approaches are required, encompassing the interface between the virome, bacteriome and mycobiome [96].

By extension, for fungal infections there is a critical question around the broader environment and in particular the aerial mycobiome as a driver for fungal infection. This has high relevance whether it be in the context of the neutropenic host with acute myeloid leukaemia, where acquisition of *A. fumigatus* from the hospital environment has been clearly documented to cause infection [97–100], or allergic fungal airway diseases where further work is required to understand the relationship between population-level sensitization to allergenic fungi and exposure to these fungi in the environment [101, 102]. Groundbreaking metagenomic approaches have been developed to characterize the aerial mycobiome that hold great promise to allow better understanding as well as prediction of the environmental factors driving respiratory fungal disease [30].

Within the context of the immunocompromised host a key issue is around how to use diagnostics to guide treatment. In this regard, the primary questions revolve around whether universal prophylaxis (for instance posaconazole in neutropenic acute myeloid leukaemia) versus pre-emptive mycological biomarker-driven therapy or directed therapy for confirmed respiratory fungal disease is most appropriate [103]. Whilst universal prophylaxis appears an expedient solution the rising incidence of fungal resistance to antimicrobials argues against such an approach [104, 105]. In contrast, directed therapy, which is useful to minimize unnecessary antifungal usage, runs the risk of late diagnosis and consequently poorer outcomes. However, these approaches have rarely been systematically compared in randomized controlled trials [106, 107].

The emergence of antifungal resistance as a major clinical issue is an area of great concern [29]. This has been a huge problem for *Candida* spp. with replacement of *C. albicans* as the dominant pathogen with other species such as *C. glabrata* and *C. krusei* in high-risk azole-exposed populations [108], as well as the global emergence of *C. auris* as a high-transmission, multidrug-resistant human opportunistic pathogen [109–111]. More recently our understanding of emergent triazole resistance in *A. fumigatus* as a consequence of both in-host adaptation to selective drug pressure as well as fungicide use in the environment [104, 105], and the observation of in-host evolution of fluconazole resistance in *C. neoformans* [112] means that we urgently need clinically robust molecular and phenotypic diagnostics to define the epidemiology and extent of resistance in clinical settings for all three major human fungal pathogens. Significant progress has been made with population-level next generation sequencing; integration of these approaches into clinical laboratory workflows as soon as possible is now a major goal.

### Immunodiagnosis of Fungal Inflammation

Immunodiagnosis is a cornerstone for the diagnosis of respiratory fungal disease such as chronic pulmonary aspergillosis, allergic bronchopulmonary aspergillosis and severe asthma with fungal sensitization, as well as having utility for the identification of fungal drivers of hypersensitivity pneumonitis [113]. The primary modality is by detection of antibody responses to immunodominant fungal antigens and allergens [114–116]. However, the field is vastly complex, because fungi have one of the highest numbers of antigenic molecules when compared to other allergens [117, 118]. In the context of aspergillosis this is of particular interest because whilst some antigens are immunodominant and therefore have utility as components of sensitive screening assays, other antigens may have higher predictive value for disease severity and progression [119–121]. For instance there are currently 23 WHO-defined fungal allergens for *A. fumigatus* (http://allergen.org). There has been some recent progress in the development of multiplex assays that can identify broad antigen repertoires for these diseases [93, 122]. Such an approach is likely to have utility for precision diagnostic medicine across chronic respiratory fungal infection, allergic fungal airway disease and hypersensitivity pneumonitis. Further progress has been made through the development of cellular response assays with a particular area of focus being fungal-reactive T cells [123–125]. These have been shown to have utility as assays to identify active infection both in the context of invasive aspergillosis as well as cystic fibrosis-related aspergillosis [126]. Large multicentre studies are required to further validate their utility for the early and accurate identification of individuals with respiratory fungal diseases. Such T cell response assays ought to be applicable to a wider range of respiratory fungal pathogens; utility has been shown already for *Aspergillus* spp. as well as *Mucor* spp [127]. Basophil activation assays, which are used to confirm the functional ability of allergens to induce effector cell degranulation, have also been shown to be useful to assess the response of patients with allergic fungal airway disease to immunotherapies such as the IgE-depleting monoclonal omalizumab [128].

### Identification of Fungal Immunogenetic Risk

Immunogenetic risk prediction for respiratory fungal disease has great promise as a key diagnostic tool in the at-risk host. Important work in this area has been undertaken within the context of primary immunodeficiencies, for instance Dectin-1/Card-9/JakStat mutations and risk of chronic mucocutaneous candidiasis [129–132]. However, there has been tremendous progress in identifying immunogenetic alleles that confer risk for invasive fungal disease in the context of transplantation, where it has been shown that either donor or recipient alleles may be implicated. In the context of haematological stem cell transplant, current data suggest that where the recipient immunogenotype is predictive of risk, this is due to defects of the recipient respiratory epithelial deficiencies, whereas where the donor immunogenotype is predictive, this maps to the donor stem cell myeloid compartment [133–136]. Extension of such approaches to other at-risk groups for respiratory fungal diseases would be of great interest, with some studies already undertaken for allergic bronchopulmonary aspergillosis for instance [137, 138]. Furthermore, most immunogenetic studies thus far have been focused on selected groups of alleles. Large-scale and multinational GWAS studies would give greater resolution to which alleles are dominant and whether they penetrate in all populations.

### Personalized Medicine to Identify Clinically Relevant Endotypes

The identification of specific clinically relevant disease endotypes has been greatly accelerated in respiratory medicine through the advent of systematic immunophenotyping studies [95, 139–142]. Given the current revolution in monoclonal antibody therapies in clinical medicine, with many agents now licensed for therapy of asthma and allergic rhinitis, repurposing of these monoclonals for allergic fungal airway diseases in particular, and better understanding of which may be more efficacious in this setting, is a current high priority [143, 144]. We are already using omalizumab routinely for IgE depletion in allergic bronchopulmonary aspergillosis and severe asthma with fungal sensitization, with accumulating but mainly case series-based clinical data to support this approach [145–147]. Mepolizumab-based targeting of eosinophilic responses is also now commonplace, although there are some safety concerns more generally as eosinophils may have a protective role against invasive aspergillosis [148, 149]. Better understanding is therefore required of which immune pathways are most contributory to progressive decline in allergic fungal airway diseases in order to enable the precise targeting of monoclonal therapeutics. In order to do this, large multicentre prospective immunoprofiling studies are required adopting a systematic approach to identify those targetable immune pathways that are most predictive of severe endotypes of allergic fungal airway disease. Optimal resolution of endotypes is achieved when systemic immune signatures (i.e. peripheral blood multiparameter flow sorting), local airway signatures (i.e. sputum transcriptome, sputum mycobiome) data and clinical data (i.e. radiological scoring, respiratory physiology and clinical questionnaires) are integrated to provide multidimensional data linked to clinical longitudinal outcomes. Such an approach has been used to identify new asthma endotypes and would be of great utility for chronic respiratory fungal diseases, where we still do not fully understand for instance why only some patients with allergic bronchopulmonary aspergillosis respond to steroids, whereas others may show a response to antifungals [150]. The emergence of artificial intelligence, or at least machine learning, has opened the door for novel approaches to identification of respiratory disease radiological endotypes, where a range of fungal-specific or at least predictive features such as nodules or cavitation exist, but could be further refined and automatically identified using non-partisan image analysis approaches [151].

## Conclusions

Over the last decade there has been greater awareness of the prevalence of respiratory fungal diseases globally. The advent of the 4th industrial revolution leaves us poised to exploit molecular engineering and post-genomic technologies and advances in combination with big data science and artificial intelligence to revolution our understanding of the pathogenesis of these complex infections. Substantial progress in drug discovery and development, as well as the rational design of novel immunotherapeutics, has greatly broadened the therapeutic armamentarium with which to combat respiratory fungal disease. The major challenge we face to translate these advances for the benefit of patients will be to ensure that these advances in the understanding of disease pathogenesis and novel therapeutic options are integrated within a personalized medicine framework to ensure the right patient gets the right treatment at the right time.
